# Beneficial Effects of Aminoguanidine on Skin Flap Survival in Diabetic Rats

**DOI:** 10.1155/2012/721256

**Published:** 2012-12-13

**Authors:** Ayse Ozturk, Cemal Fırat, Hakan Parlakpınar, Aysun Bay-Karabulut, Hale Kirimlioglu, Ali Gurlek

**Affiliations:** ^1^Department of Plastic Reconstructive and Aesthetic Surgery, Inonu University School of Medicine, 44380 Malatya, Turkey; ^2^Department of Plastic, Reconstructive and Aesthetic Surgery, Turgut Ozal Medical Center, Inonu University School of Medicine, 44380 Malatya, Turkey; ^3^Department of Pharmacology, Inonu University School of Medicine, 44380 Malatya, Turkey; ^4^Department of Biochemistry, Inonu University School of Medicine, 44380 Malatya, Turkey; ^5^Department of Pathology, Inonu University School of Medicine, 44380 Malatya, Turkey

## Abstract

Random flaps in DM patients have poor reliability for wound coverage, and flap loss remains a complex challenge. The protective effects of aminoguanidine (AG) administration on the survival of dorsal random flaps and oxidative stress were studied in diabetic rats. Two months after the onset of DM, dorsal McFarlane flaps were raised. Forty rats were divided into four groups: (1) control, (2) AG, (3) DM, and (4) DM + AG groups. Flap viability, determined with the planimetric method, and free-radical measurements were investigated. In addition, HbA1c and blood glucose levels, body weight measurements, and histopathological examinations were evaluated. The mean flap necrotic areas (%) in Groups I to IV were 50.9 ± 13.0, 32.9 ± 12.5, 65.2 ± 11.5, and 43.5 ± 14.7, respectively. The malondialdehyde (MDA) and nitric oxide (NO) levels were higher in the DM group than in the nondiabetic group, while the reduced glutathione (GSH) levels and superoxide dismutase (SOD) activity were reduced as a result of flap injury. In the diabetic and nondiabetic groups, AG administration significantly reduced the MDA and NO levels and significantly increased GSH content and SOD enzyme activity. We concluded that AG plays an important role in preventing random pattern flap necrosis.

## 1. Introduction 

Diabetes mellitus (DM) is characterized by a series of complications that affect many organs. Due to long-term incubation with glucose, increments in the chemical modification of proteins lead to the formation of heterogenous, toxic, and antigenic glycation end products (AGEs) [1]. Hyperglycation is thought to increase oxidative stress. Hence, glycation and oxidation appear to be inextricably linked to each other. Many examples of enhanced lipid peroxidation and malondialdehyde (MDA) accumulation are present in patients with DM [2].

Research has shown that structural and functional impairments in the microcirculation of diabetic skin reduce skin perfusion [3, 4]. Partial or total necrosis of skin flaps remains a significant problem in plastic and reconstructive surgery. The main causes of necrosis are classified as inadequate arterial inflow, insufficient venous outflow, or both [5, 6]. These factors lead to ischemia that frequently occurs in the most distal portion of the flap. Experimental work has shown that augmenting the vascularity of an ischemic skin flap can significantly improve flap survival [7, 8]. The clinical use of random pattern flaps may be limited by the development of necrosis in the distal area because of ischemia-reperfusion (I/R) injury. The effects of I/R on random pattern skin flaps have been documented in numerous species of experimental animals. The leading mechanism of this toxicity is believed to involve reactive oxygen radical species (ROS) generation and neutrophil accumulation [3, 8]. Extensive research regarding enhancing skin flap viability with pharmacological manipulation has been performed [9]. However, the pathophysiological processes underlying flap ischemia and the mechanisms of pharmacological augmentation of flap survival remain unclear. New molecular biological insights offer possibilities for a better understanding of flap survival and necrosis. Surgical delay, which enhances flap viability, is an effective technique often used for this purpose [10]. However, this approach has the disadvantage of involving a two-stage procedure. Considering the role of free radicals in flap necrosis, the perioperative application of a free-radical scavenger in a random pattern flap model in diabetic rats will theoretically increase flap survival by improving functional deficits in the microcirculation and by reducing free-radical-induced damage to the flaps. We hypothesized that acute preoperative application of AG would not affect the vascular structure, but rather act by scavenging free radicals in the tissue, as well as improving microcirculation.

Aminoguanidine (AG) is a compound that selectively and competitively inhibits inducible nitric oxide synthase (iNOS). The inhibition of iNOS in turn leads to a decrease in nitric oxide (NO) generation in tissues [11]. AG is also known to prevent advanced glycation by inhibiting diamine oxidase binding [12, 13]. Also, AG has been shown to inhibit glucose-mediated tissue damage by trapping the reactive carbonyl species and minimizing complications of DM [11]. Despite the vast number of clinical studies comparing various reconstructive methods in DM, the number of experimental studies investigating flap survival and the effect of antioxidant treatment are limited. No report regarding the effects of AG on diabetes-induced oxidative stress in the skin flap has been reported. Therefore, in this study we investigated the effects of AG on preventing secondary complications of DM and the survival of dorsal random flaps in diabetic and nondiabetic rats.

## 2. Materials and Methods

### 2.1. Experimental Protocol and Diabetes Rat Model

All experiments in this study were performed in accordance with the guidelines for animal research from the National Institutes of Health and the Committee on Animal Research at our institution. In this study, 40 female Wistar rats weighing 200–250 g were used. They were maintained under a 12–12 h light-dark cycle at 21 ± 2°C with food and water freely available except during the time of the experiments. The rats were preoperatively anesthetized with an intraperitoneal (i.p.) application of a mixture consisting of ketamine hydrochloride (75 mg/kg) and xylazine hydrochloride (8 mg/kg). 

Diabetes was induced in rats by a single i.p. injection of streptozotocin (STZ) (45 mg/kg). STZ (Sigma Chemical Co., St. Louis, MO, USA) was dissolved in 0.1 M citrate buffer; pH 4.5. Normal rats were injected with the equivalent volume of citrate buffer. Plasma glucose levels were measured with a semiautomatic glucose analyzer (Bayer, RA50) using the glucose oxidase method, on the 1st day (before STZ induction), the 5th day (after STZ administration and before flap elevation), and the 7th day (after flap elevation) in all groups. After a blood sample was obtained through the tail vein, animals with glucose levels higher than 250 mg/dL on the 5th day after STZ was administered were included in the study. No insulin was administered to the animals. 

### 2.2. Treatment Groups and Followup

40 rats were randomly divided into four groups. 10 of them died during the experimental study because of diabetes mellitus and surgical complications. Therefore, the dead rats were excluded from the study. The number of the groups is as follows.

 Group 1 (*n* = 10) (nondiabetic, control group) was treated with vehicle only (i.p.).
 Group 2 (*n* = 8) (nondiabetic group treated with AG) was treated with 100 mg/kg (i.p.) aminoguanidine (AG) (Sigma-Aldrich Chemie Gmbh, Steinheim, Germany) 1 h before skin flap surgery; the AG treatment continued for 6 days after flap elevation (*n* = 8; the number of exitus rats: 2).
 Group 3 (*n* = 6) (diabetic group, no treatment) was treated with vehicle only (*n* = 6; the number of exitus rats: 4).
 Group 4 (*n* = 6) (diabetic group treated with AG) was treated with 100 mg/kg AG 1 h before skin flap surgery. The AG treatment continued for 6 days after flap elevation (*n* = 6; the number of exitus rats: 4).
 The dosage of AG (100 mg/kg) was selected according to the levels used successfully in experimental models for iNOS blockage and antioxidant properties [14, 15].

### 2.3. Flap Elevation Procedure

**Figure 1:**
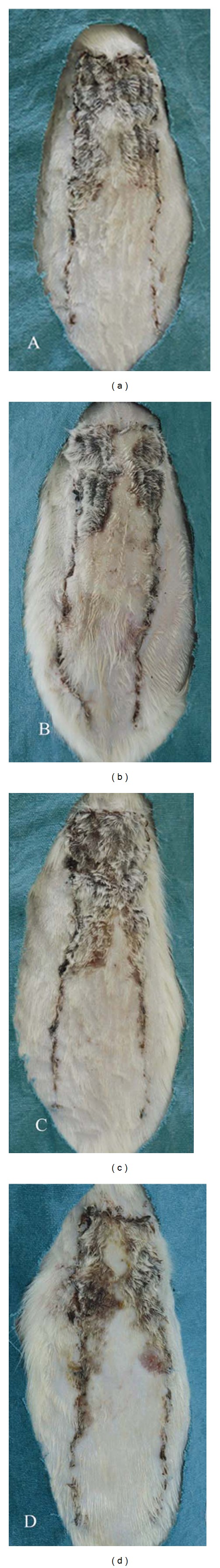
Flap necrosis area in the groups.

**Figure 2:**
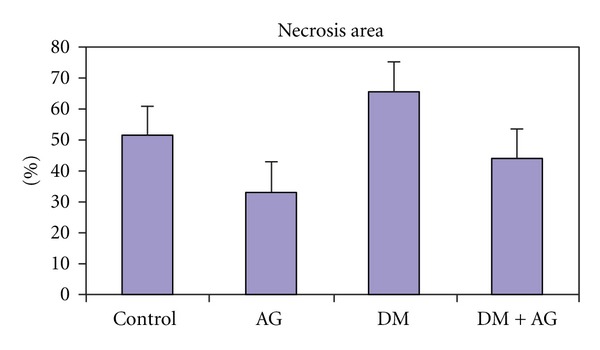
Appearance of rat dorsal random pattern flaps 7 days after surgery, (a) control group, (b) AG group, (c) DM group, and (d) DM + AG group. The photographs show samples of flap necrosis and survival areas in the corresponding groups.

The backs of the rats were completely shaved with an electrical clipper and then sterilized for surgery with Betadine (Poviiodeks, Kim-Pa Corporation, Istanbul, Turkey). During the surgical procedure, aseptic conditions were maintained by providing a local sterile environment. A dorsal random pattern skin flap 10 × 3 cm was elevated on the dorsal trunk of the rats according to the method described by Khouri et al. [16] with meticulous homeostasis. Then the flap was sutured back into place with 5/0 running nylon sutures (Figure 2). All rats were housed individually during the postoperative period to prevent cannibalism. Flap viability was evaluated on the 7th day, after the initial operation (flap elevation), at which time a certain amount of necrosis in the distal part of all dorsal flaps was noted (Figure 1). The flap survival and necrosis areas were assessed with inspection, when the demarcation between the eschar and viable skin was clear. All groups were photographed, the necrotic skin (defined by the necrotic skin borders) and total flap (defined by the surgical borders) areas were delineated, and the surface areas were calculated (in square centimeters) using computer-assisted planimetry. The necrotic surface area was divided by the total flap area, and the results are expressed as percentages of skin necrosis (Figure 1). The animals were then sacrificed under general anesthesia. The skin biopsy was harvested from an area between 3 and 4 cm proximal of the flap to determine the malondialdehyde (MDA), nitric oxide (NO), and reduced glutathione (GSH) levels, and superoxide dismutase (SOD) activities.

### 2.4. Biochemical Determinations

Two hundred milligrams of frozen flap tissue biopsy specimens, cut into pieces on dry ice, were homogenized in 1.15% KCl buffer (1 : 9, w/v) using a manual glass homogenizer for approximately 5 min and flushed with centrifugation for approximately 10 s to remove large debris. The supernatant was used for analysis. 

MDA in tissues was determined with Uchiyama and Mihara's method [17]. A 3 mL aliquot of 1% phosphoric acid and 1 mL of 0.6% thiobarbituric acid solution were added to 0.5 mL of 10% tissue homogenate pipetted into a tube. The mixture was heated in boiling water for 45 min. After cooling, the color was extracted into 4 mL of n-butanol. The absorbance was measured in a spectrophotometer (Ultraspec Plus, Pharmacia LKB Biochrom, Cambridge, UK) with 535 and 525 nm. The lipid peroxide amounts were calculated as thiobarbituric acid reactive substances (TBARS) of lipid peroxidation and are given as nmol/g tissue.

Since tissue nitrite (NO_2_^−^) and nitrate (NO_3_^−^) levels can be used to estimate nitric oxide (NO) production, we measured the concentration of these stable NO oxidative metabolites. Quantification of (NO_2_^−^) and (NO_3_^−^) was based on the Griess reaction, in which a chromophore with a strong absorbance at 545 nm is formed by the reaction of (NO_2_^−^) with a mixture of naphthlethylenediamine and sulfanilamide [14]. Results are expressed as nmol/g tissue. 

Reduced GSH was determined with the spectrophotometric method, which was based on the use of Ellman's reagent [18]. Results are expressed as nmol/mg tissue.

SOD activity was determined with Sun et al.'s method [19] by inhibiting nitroblue tetrazolium (NBT) reduction with xanthine/xanthine oxidase used as a superoxide generator. One unit of SOD is defined as the amount of protein that inhibits the rate of NBT reduction by 50%. Results are expressed as U/g protein.

### 2.5. Histopathological Evaluation

In a 2-month period, diabetes-induced changes were observed in the vessels of diabetic skin. The specimens were fixed in 10 % formaldehyde, and routine procedures were performed. Paraffin sections were cut including the line between the necrotic area and the viable area. The slides were then stained with hematoxylin and eosin (H&E) and Masson's trichrome stain. In addition, body weight measurements and HbA1c levels were investigated to evaluate chronic changes in DM. 

### 2.6. Statistical Analysis

Data are expressed as means and standard deviations. All statistical analyses were conducted using SPSS statistical software (SPSS, version 15.0, for Windows, SPSS Inc., Chicago, IL). Flap tissue MDA, NO, GSH, and SOD levels, necrotic area, blood glucose, body weight, and glycated hemoglobin measurements were analyzed with one-way analysis of variance (ANOVA). Either the post hoc Tukey HSD test (for homogeneous variances) or Tamhane's T2 test (for non-homogeneous variances) was performed for multiple comparisons. The comparison between groups was performed with the Mann-Whitney *U* test. *P* values less than 0.05 were regarded as statistically significant.

## 3. Results

The infarct size/flap size ratio was analyzed in each sample (Figures 1 and 2 and Table 1). The regions of survival and the necrotic areas were clearly demarcated in every flap since the flaps did not shrink on the postoperative 7th day. The surviving skin appeared pink-white, tender, and normal in texture and bled when cut, whereas the necrotic skin was black and rigid and did not bleed.

The MDA, NO, GSH, and SOD levels obtained from the skin tissues of rats in all groups were recorded (Table 1). The MDA and NO levels were higher in the diabetic group than the nondiabetic group, while the GSH levels and SOD activities were reduced because of tissue injury. AG administration significantly reduced the MDA and NO levels and significantly increased the GSH content and SOD enzyme activities in the diabetic and nondiabetic groups.

In the DM (Groups 3-4) groups, the blood glucose levels were higher than the control and AG-treated groups (Tables 2-3). In the current study, to investigate the chronic changes in DM, the HbA1c levels were also studied. HbA1c shows the regulation of blood glucose in the most recent 2-3 months, and high levels of HbA1c indicate bad regulation of DM. In our study, in the rats after 2 months of induction of DM, the HbA1c levels were significantly increased when compared to the other groups together in which no antidiabetics were administered (Tables 2-3). 

In the AG group, the number of hair follicles (MT ×100) and the cytological appearance of the fibroblasts (MT ×400) were not significantly different when compared with the control group. In the DM group, a significant decrease in the number of hair follicles (MT ×100) and dermal atrophy features including reduced dermal thickness, cytologically more rounded appearance of fibroblasts (MT ×400), and slightly irregular collagen structure was observed when compared with the control group. 

**Figure 3:**
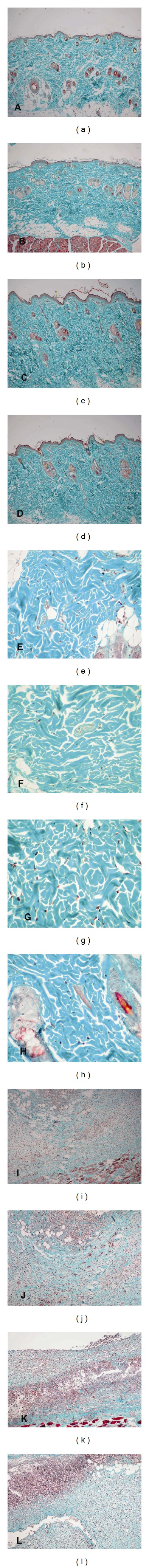
Light microscopy of the skin flaps (Masson's trichrome stain). Control group (a, e, i), AG group (b, f, j), DM group (c, g, k), DM + AG group (d, h, l). Comparison of hair follicles among the four groups (a, b, c, d), comparison of fibroblasts among the four groups (e, f, g, h), comparison of inflammation among the four groups (i, j, k, l). In the AG group (b, f), in the number of hair follicles (MT ×100) and in the cytological appearance of the fibroblasts (MT ×400), no significant difference was observed when compared to the control group (a, e). In the DM group (c), a significant decrease in the number of hair follicles (MT ×100) and dermal atrophy features including reduced dermal thickness, cytologically more rounded appearance of fibroblasts (MT ×400), and slightly irregular collagen structure was observed when compared to the control group (e). Inflammation and necrosis were more apparent in the DM groups (k, l) (MT ×100).

Inflammation and necrosis were more apparent in the DM groups (MT ×100) (Figure 3). Body weight was lower in the DM groups, when compared with the control group (Tables 2-3).

The diabetic group exhibited a significant increase in necrotic areas expressed as the percentage of the risk zone (65.2 ± 11.5) when compared with the nondiabetic groups (50.9 ± 13.0). AG administration resulted in a significant decrease in skin flap necrosis in the diabetic (43.5 ± 14.7) and nondiabetic groups (32.9 ± 12.5). 

## 4. Discussion 

In the current study, we focused on investigating the effects of AG, a potent scavenger of free radicals and an antioxidant, on the survival of random pattern skin flaps in diabetic rats. For this reason, we measured the levels of MDA (end product of lipid peroxidation), NO, GSH content, and SOD activity. We also calculated flap survival with the computer-assisted planimetric method and measured blood glucose levels, body weight, and glycated hemoglobin. The rat dorsal skin flap model was originally described by McFarlane et al. [20] to study skin flap necrosis and its prevention. The survival pattern of this flap model is different from that of the skin graft [21, 22]. Here, we used the modified 3 × 10 cm caudal-based dorsal flap model, which is perfused by two constant sacral axial vessels [23]. 

In this study, flap elevation significantly increased the MDA and NO levels especially in the diabetics group. AG administration significantly reduced the MDA and NO levels in the control and diabetic rats. The beneficial effect of AG is probably its antioxidant, free-radical scavenger, and protective lipid peroxidation effects; however, the mechanism is not clear. AG may directly eliminate free oxygen radicals such as peroxynitrite or directly increase antioxidant enzyme activity and prevent inhibition of these enzymes. Our results with AG pretreatments confirm the findings of Jakus et al. [24] and Parlakpinar et al. [25] and have demonstrated that AG has the potential to inhibit MDA. For the contribution of free-radical generation and in turn oxidative stress, lipid peroxidation and altered levels of some endogenous scavengers are taken as indirect in vivo reliable indices [26]. Researchers have demonstrated that depletion of GSH precedes the induction of lipid peroxidation [26]. The increase in lipid peroxidation products, in clinical and experimental diabetes, is an important result of oxygen-derived free radical stress. These products may be important in the pathogenesis of vascular complications in DM.

NO is an inorganic free radical gas that plays an important role in numerous biochemical processes. Among these, NO is involved in the regulation of vascular tone and organ blood flow and also in the inhibition of platelet and neutrophil aggregation [14]. 

Previous studies have demonstrated that the development of diabetic complications in DM is closely related to the increased generation of superoxide anion (O_2_^−^) and NO [27]. NO can be destructive and protective, which may be attributable to the different oxidation-reduction states of the molecule [28]. It may also be cytotoxic, leading to the formation of tissue-damaging free radicals such as peroxynitrite and subsequently hydroxyl radicals [29]. All of these circumstances exert inadequate blood vessel profusion, which is the major reason of the flap necrosis. The beneficial actions of AG probably derive from its inhibitor effects on the formation of highly reactive advanced AGEs associated with the pathogenesis of secondary complications of diabetes. Moreover, AG ameliorates various complications of diabetes and prevents age-related arterial stiffening through inhibiting AGE formation. AG also inhibits NO synthase, in particular the inducible NO synthase isoform. The inducible NO synthase isoform is associated with the production of large quantities of NO synthase in response to cytokines, for example [30]. Hasan et al. [31] reported that AG strongly inhibited cytokine-induced iNOS, while vascular constitutive isoform of NO synthesis, responsible for vascular control, was only weakly inhibited by AG.

Micro- and macrovascular complications of DM cause significant morbidity and mortality. Despite all efforts to use well-vascularized tissues and axial flaps in reconstructing defects in diabetics, random flaps remain an important alternative. Clinical experience with the use of random flaps, especially in the lower extremity, has shown poor reliability for wound coverage [23, 32]. Free radicals damage the integrity of the microvascular architecture and cause lipid peroxidation in the cell membrane, all resulting in tissue necrosis [33].

In accordance with our results, researchers have reported that diabetic patients have significant defects in antioxidant protection and generation of ROS, which may play an important role in the etiology of diabetic complications [22]. A decrease in SOD, catalase (CAT), peroxidase (Px), ceruloplasmin (Cp), and glutathione peroxidase (GSH-Px) activities as well as a decrease in the GSH level and an increase in the concentration of glutathione disulfide (GSSG) were observed in erythrocytes of diabetic patients and in tissues from diabetic animals [34]. Masuyer et al. [35] demonstrated that AG was a free radical scavenger against superoxide and hydroxyl radicals. These data are consistent with our results that AG administration reduced the MDA and NO levels and increased GSH levels and SOD enzyme activities.

We found that the effect of flap elevation and diabetes significantly reduced the GSH level and SOD enzyme activities in the control group. AG administration had a significantly beneficial effect on these parameters (Table 1). Considering the reduced oxidative damage due to the AG treatment, all investigators attributed protective actions of AG to its antioxidative, free radical scavenger, and preventive ROS formation activity. 

AG administration significantly reduced the ratio of skin flap necrosis, which was determined with planimetry and photography (Figures 1 and 2, Table 1). According to our findings, the level of free radicals in the flap tissue was inversely correlated with the survival area. For instance, the necrosis rate and the free radical concentration were greatest in the DM group (Group 3). The ischemia in the random flap, which was expected to be the greatest in the DM group due to microangiopathy, increased the oxidative stress even further. However, in the AG-treated groups, the necrosis rate and free-radical concentrations were significantly reduced when compared with the noncontrolled diabetics.

We studied the HbA1c levels and body weight measurements to investigate chronic changes in DM. HbA1c shows the regulation of blood glucose in the latest 2-3 months, and high levels of HbA1c show bad regulation DM. In our study, after 2 months of induction of DM in rats, the HbA1c levels were significantly increased [21]. In the DM (Groups 3-4) groups, the blood glucose levels were higher than in the control and AG groups (Tables 2-3). The body weights were lower in the DM groups, when compared with the control group. The weight loss in our study, particularly in the type 1 diabetic rats, was in accordance with the literature [21]. 

The use of random flaps in the diabetic population has been approached with pessimism, generally as a result of the high failure rate. This failure may be due to poor survival of the flap, general ischemia of the surrounding tissues, and the problems of wound healing. Our results suggested the following: (1) dorsal random flap necrosis in rats is correlated with the concentration of free radicals in the tissue. (2) Diabetic flaps show a greater amount of necrosis and increased the tissue free radical concentration. (3) Perioperative application of AG decreases free-radical concentration and increases flap survival. 

In conclusion, we believe that AG has beneficial effects in improving skin flap viability, especially when distal flap necrosis is a potential complication of longer flaps in diabetic patients. Apart from an antioxidant and free radical scavenging effects of AG, AG acts on both NO and histamine enzyme systems. It is well documented that in mammals, histamine is oxidated principally by histamine N-methyltransferase (HNMT) and DAO. DAO, through elimination of histamine from the tissues and bloodstream, is very important for the recovery from mast cell-linked reactions. It has been shown that dioxin stimulates the synthesis and secretion of IgE-dependent histamine-releasing factor in mouse cells as well [36]. Dioxin stimulates synthesis and secretion of IgE-dependent histamine-releasing factor. Despite the benefits of acute preoperative administration of AG, its long-term usage with larger samples and groups including insulin given animals should be investigated with further studies. 

## Figures and Tables

**Table 1:** Necrosis area of groups (%) ± standard deviation (SD) and MDA, NO, GSH, and SOD activity levels of the flap tissue and *P* ≤ 0.05 values.

Groups	Necrosis area (%)	MDA (nmol/g tissue)	NO (nmol/g tissue)	SOD (U/g protein)	GSH (nmol/g tissue)
(I) Control	50,9 ± 0,13	25,14 ± 12,92	150,19 ± 18,66	4,66 ± 1,72	0,96 ± 0,11
(II) AG	32,9 ± 0,12	21,99 ± 10,05	120,93 ± 10,85	6,80 ± 1,30	1,31 ± 0,20
(III) DM	65,2 ± 0,11	100,74 ± 83,19	203,75 ± 11,26	2,72 ± 0,47	0,68 ± 0,08
(IV) DM + AG	43,5 ± 0,14	45,22 ± 27,20	159,58 ± 16,00	5,75 ± 0,96	0,90 ± 0,11

Comparisons of the groups	*P* values*				

I-II	0.001		0.001	0.009	0.001
I–III	0.001	0.009	0.001	0.003	0.001
I–IV		0.023		0.050	
II-III	0.001	0.005	0.002	0.002	0.002
II–IV		0.020	0.003		0.005
III-IV	0.003		0.004	0.004	0.010

*Statistically significant values.

**Table 2:** Measured glucose and HbA1c levels and body weight in the groups.

Groups	Glucose (mg/dL)	HbA1c (%)	Body weight (g)
Group I: Control			

First day (before STZ administration)	145,76 ± 39,26	3,80 ± 0,16	206,23 ± 54,22
5th day (5 days after STZ administration)	140,23 ± 31,66	3,78 ± 0,12	199,30 ± 50,96
Before flap elevation	174,00 ± 30,75	3,95 ± 0,26	197,00 ± 12,98
7th day (7 days after flap elevation)	145,69 ± 35,87	4,21 ± 0,39	195,23 ± 17,09

Group II: AG			

First day (before STZ administration)	135,12 ± 43,36	3,92 ± 0,26	210,37 ± 52,08
5th day (5 days after STZ administration)	136,00 ± 41,82	3,93 ± 0,26	209,12 ± 50,31
Before flap elevation	153,37 ± 24,64	3,93 ± 0,64	194,87 ± 38,84
7th day (7 days after flap elevation)	149,12 ± 28,72	4,50 ± 0,63	192,75 ± 38,46

Group III: DM			

First day (before STZ administration)	125,00 ± 30,43	3,93 ± 0,29	208,83 ± 52,8
5th day (5 days after STZ administration)	395,83 ± 79,83	5,30 ± 0,55	192,16 ± 50,02
Before flap elevation	306,33 ± 101,79	8,30 ± 1,46	182,16 ± 43,23
7th day (7 days after flap elevation)	450,83 ± 165,73	9,45 ± 2,50	170,16 ± 42,70

Group IV: DM + AG			

First day (before STZ administration)	153,16 ± 45,10	3,91 ± 0,29	207,83 ± 53,03
5th day (5 days after STZ administration)	382,83 ± 51,54	5,30 ± 0,55	192,33 ± 49,65
Before flap elevation	373,00 ± 124,96	7,98 ± 1,38	188,83 ± 44,56
7th day (7 days after flap elevation)	455,16 ± 109,57	8,20 ± 0,80	187,16 ± 44,00

**Table 3:** Measured glucose and HbA1c levels and body weight in the groups and *P* ≤ 0.05 values.

Groups	HbA1c^#^	HbA1c*	HbA1^+^	Glucose^#^	Glucose*	Glucose^+^	BW^#^	BW*	BW^+^
I-II									
I–III		0.001	0.001		0.000	0.000			0.008
I–IV	0.001	0.001	0.001	0.001	0.001	0.001		0.079	
II-III	0.002	0.002	0.002	0.002	0.002	0.002	0.028		0.010
II–IV	0.002	0.002	0.002	0.002	0.002	0.002	0.020		0.052
III-IV									0.024

Groups; I: Control; II: AG; III: DM; IV: AG + DM.

^#^5th day; *Before flap elevation; ^+^After flap elevation.

## References

[1] Kennedy L, Baynes JW (1984). Non-enzymatic glycosylation and the chronic complications of diabetes: an overview. *Diabetologia*.

[2] Vlassara H, Palace MR (2003). Glycoxidation: the menace of diabetes and aging. *Mount Sinai Journal of Medicine*.

[3] Sun F, Iwaguchi K, Shudo R (1999). Change in tissue concentrations of lipid hydroperoxides, vitamin C and vitamin E in rats with streptozotocin-induced diabetes. *Clinical Science*.

[4] Thornalley PJ (2003). Use of aminoguanidine (Pimagedine) to prevent the formation of advanced glycation endproducts. *Archives of Biochemistry and Biophysics*.

[5] Katz A, Ekberg K, Johansson BL, Wahren J (2001). Diminished skin blood flow in type I diabetes: evidence for non-endothelium-dependent dysfunction. *Clinical Science*.

[6] Khan F, Elhadd TA, Greene SA, Belch JJF (2000). Impaired skin microvascular function in children, adolescents, and young adults with type I diabetes. *Diabetes Care*.

[7] Myers MB, Cherry G (1968). Causes of necrosis in pedicle flaps. *Plastic and Reconstructive Surgery*.

[8] Kerrigan CL (1983). Skin flap failure: pathophysiology. *Plastic and Reconstructive Surgery*.

[9] Pang CY, Forrest CR, Neligan PC, Lindsay WK (1986). Augmentation of blood flow in delayed random skin flaps in the pig: effect of length of delay period and angiogenesis. *Plastic and Reconstructive Surgery*.

[10] O’Toole G, MacKenzie D, Poole M, Buckley MF, Lindeman R (2001). A review of therapeutic angiogenesis and consideration of its potential applications to plastic and reconstructive surgery. *British Journal of Plastic Surgery*.

[11] Misko TP, Moore WM, Kasten TP (1993). Selective inhibition of the inducible nitric oxide synthase by aminoguanidine. *European Journal of Pharmacology*.

[12] Sugimoto K, Yagihashi S (1997). Effects of aminoguanidine on structural alterations of microvessels in peripheral nerve of streptozotocin diabetic rats. *Microvascular Research*.

[13] Tilton RG, Chang K, Hasan KS (1993). Prevention of diabetic vascular dysfunction by guanidines: inhibition of nitric oxide synthase versus advanced glycation end-product formation. *Diabetes*.

[14] Parlakpinar H, Ozer MK, Acet A (2005). Effect of aminoguanidine on ischemia-reperfusion induced myocardial injury in rats. *Molecular and Cellular Biochemistry*.

[15] Xu J, Li N, Dai DZ, Yu F, Dai Y (2008). The endothelin receptor antagonist CPU0213 is more effective than aminoguanidine to attenuate isoproterenol-induced vascular abnormality by suppressing overexpression of NADPH oxidas, ETA, ETB, and MMP9 in the vasculature. *Journal of Cardiovascular Pharmacology*.

[16] Khouri RK, Angel MF, Edstrom LE (1986). Standardizing the dorsal rat flap. *Surgical Forum*.

[17] Uchıyama M, Mıhara M (1978). Determination of malonaldehyde precursor in tissues by thiobarbituric acid test. *Analytical Biochemistry*.

[18] Ellman GL (1959). Tissue sulfhydryl groups. *Archives of Biochemistry and Biophysics*.

[19] Sun Y, Oberley LW, Li Y (1988). A simple method for clinical assay of superoxide dismutase. *Clinical Chemistry*.

[20] McFarlane RM, DeYoung G, Henry RA (1965). The design of a pedicle flap in the rat to study necrosis and its prevention. *Plastic and Reconstructive Surgery*.

[21] Kȩdziora-Kornatowska KZ, Luciak M, Błaszczyk J, Pawlak W (1998). Effect of aminoguanidine on the generation of superoxide anion and nitric oxide by peripheral blood granulocytes of rats with streptozotocin-induced diabetes. *Clinica Chimica Acta*.

[22] Opara EC (2002). Oxidative stress, micronutrients, diabetes mellitus and its complications. *Journal of The Royal Society for the Promotion of Health*.

[23] Attinger CE, Ducic I, Cooper P, Zelen CM (2002). The role of intrinsic muscle flaps of the foot for bone coverage in foot and ankle defects in diabetic and nondiabetic patients. *Plastic and Reconstructive Surgery*.

[24] Jakus V, Hrnciarova M, Carsky J (1999). Inhibition of enzymatic proteinglycation and lipid peroxidation by drugs with antioxidant activity. *Life Sciences*.

[25] Parlakpinar H, Koc M, Polat A (2004). Protective effect of aminoguanidine against nephrotoxicity induced by amikacin in rats. *Urological Research*.

[26] Velazquez E, Winocour PH, Kesteven P, Alberti KGMM, Laker MF (1991). Relation of lipid peroxides to macrovascular disease in Type 2 diabetes. *Diabetic Medicine*.

[27] Moncado S, Palmer RMJ, Higgs EA (1991). Nitric oxide: physiology, pathophysiology and pharmacology. *Pharmacological Reviews*.

[28] Lipton SA, Choi YB, Pan ZH (1993). A redox-based mechanism for the neuroprotective and neurodestructive effects of nitric oxide and related nitroso-compounds. *Nature*.

[29] Radi R, Beckman JS, Bush KM, Freeman BA (1991). Peroxynitrite oxidation of sulfhydryls: the cytotoxic potential of superoxide and nitric oxide. *Journal of Biological Chemistry*.

[30] Nilsson BO (1999). Biological effects of aminoguanidine: an update. *Inflammation Research*.

[31] Hasan K, Heesen BJ, Corbett JA (1993). Inhibition of nitric oxide formation by guanidines. *European Journal of Pharmacology*.

[32] Oishi SN, Levin LS, Pederson WC (1993). Microsurgical management of extremity wounds in diabetics with peripheral vascular disease. *Plastic and Reconstructive Surgery*.

[33] Im MJ, Manson PN, Bulkley GB, Hoopes JE (1985). Effects of superoxide dismutase and allopurinol on the survival of acute island skin flaps. *Annals of Surgery*.

[34] Abou-Seif MAM, Youssef AA (2001). Oxidative stress and male IGF-1, gonadotropin and related hormones in diabetic patients. *Clinical Chemistry and Laboratory Medicine*.

[35] Courderot-Masuyer C, Dalloz F, Maupoil V, Rochette L (1999). Antioxidant properties of aminoguanidine. *Fundamental and Clinical Pharmacology*.

[36] Oikawa K, Ohbayashi T, Mimura J (2002). Dioxin stimulates synthesis and secretion of IgE-dependent histamine-releasing factor. *Biochemical and Biophysical Research Communications*.

